# The sagitta in 3D reconstruction of linea alba on routine CT scans is predictive of postoperative burst abdomen

**DOI:** 10.1007/s10029-025-03303-0

**Published:** 2025-03-12

**Authors:** Matthias Mehdorn, Benedikt Schnarkowski, Sigmar Stelzner, Uwe Scheuermann, Woubet Tefera Kassahun, Timm Denecke, Stefan Niebisch, Hans-Jonas Meyer

**Affiliations:** Department of Visceral, Transplant, Thoracic and Vascular Surgery, University Hospital of Leipzig, Liebigstraße 20, 04103 Leipzig, Germany

**Keywords:** Linea alba, 3D morphometrics, Sagitta, Burst abdomen

## Abstract

**Purpose:**

Burst abdomen (BA) is a relevant complication after abdominal surgery that causes additional surgical procedures, prolonged hospital stays and long-term morbidity. Several underlying risk factors exist and have been characterized previously. Those risk factors consist of surgical and medical factors. Recently, CT-derived body composition is of rising interest and 3D reconstruction of the linea alba has been studied. The clinical significance of those parameters is not clear. We therefore performed an analysis of linea alba 3D reconstruction measurements and their prognostic significance on the development of BA.

**Methods:**

An institutional data base of patients with post operative wound infections was assembled. The subgroup of patients with BA was compared to controls. If the patients had complete preoperative abdominal CT scans, their images were further analyzed and 3D reconstruction of the linea alba was performed. Subsequently, lineal alba was measured at predetermined positions. Those values were evaluated as risk factors for postoperative BA.

**Results:**

A total of 72 patients with BA and 32 controls were eligible for the analysis. We found body mass index-related significant differences as well as sex related differences in linea alba width. Furthermore, BA patients had a significantly wider linea alba and longer sagitta compared to controls. In the multivariate analysis of linea alba measurements and clinical parameters, the length of the sagitta was significantly associated with the risk of BA (OR 1.266; 95% CI 1.011–1.585; *p* = 0.04).

**Conclusion:**

In this study of 3D reconstruction of the linea alba from routine CT scans, we could show that a longer sagitta was associated with an increased risk of postoperative BA.

## Introduction

The linea alba is the junction of both rectus sheaths and forms the abdominal midline. It is the target area of median laparotomies as access to the abdominal cavity during open abdominal surgery. At the end of the surgery, the linea alba is reconstituted by sutures to close the abdominal cavity. Incisional hernias are present in one third of the patients as long-term complications after open abdominal surgery. Short-term complications of the abdominal wall can also occur in the form of abdominal wall dehiscence, also called burst abdomen (BA). This acute complication often requires urgent repair and may cause relevant morbidity with slowly healing large wounds and even increased mortality. Risk factors for BA are liver cirrhosis [1, 2], emergency surgery [1–3]. Current risk scores for postoperative fascial dehiscence include chronic obstructive pulmonary disease, postoperative coughing [2–4] or surgical site infections [3, 5] among others. Additionally, obesity determined by the body mass index (BMI) could be established as additional risk factor [4, 5], but the BMI does not necessarily reflect the amount of abdominal obesity. Instead, abdominal obesity can objectively be evaluated from routine CT scans and could be identified as risk factor for burst abdomen [6].

In general, the width of the linea alba varies from the xiphoidal process down to the pelvic bones. Cut-off values, derived from morphometric studies, have defined the diastasis recti. The normal width of the linea alba was shown to be around two centimeters in CT imaging [7]. Accordingly, the European Hernia society defined diastasis recti as linea alba wider than two centimeters, admitting no correlation between linea alba width and symptoms such as local pain [8]. However, the evaluation of routine CT scans relativized those studies and concluded that a widened linea alba can be found in most of the population without any clinical significance, thus questioning the previous definitions of pathologic cut-off values [9]. Still, those studies underline the possibility of using routine CT scans for evaluation of clinical features connected to the abdominal wall.

Recently, morphometric studies from routine CT scans including a 3D reconstruction of the linea alba have been published as a descriptive work [10], pointing out gender, age and body mass index (BMI) dependent width of the linea alba. A large variability of the measurements along the linea alba was described. As this was a descriptive study, no clinical message was included. They found out that the sagitta of the linea alba correlated with the body mass index and the total abdominal and visceral (fat) area. The sagitta represents the curvature of the linea alba. We recently found out, that visceral obesity, determined as distance between lumbar vertebra and linea alba, predicted the risk of burst abdomen, hypothesizing a relation between abdominal diameter and subsequent fascial tension [6].

The present study was carried out to assess the observations from the previous study by Gueroult and colleagues [10] and to evaluate the implications of CT-derived linea alba 3D- morphometrics for the prediction of postoperative burst abdomen.

## Materials and methods

The study was approved by the local Ethics review board and retrospectively registered in the German register for clinical trials.

The register was set up to analyze patients from our department that had received an abdominal surgery and developed a surgical site infection in the years 2015–2018. The data acquisition was performed in a retrospective manner using electronic patient charts, filtered by the ICD-10 code for wound infection (T81.4). Patients were included if a surgical site infection in accordance with the center of disease control and prevention definition of a surgical site infection event [11] was diagnosed between the fascial level and the skin. Consequently, demographic as well as surgical, medical and data on the postoperative course were assembled. Patients with incomplete datasets or who were admitted to our department after previous surgery in another hospital were excluded.

For the present analysis only patients with median laparotomies were selected, as this is the are studied on CT scans. Hence, patients with transverse incisions were excluded.

The surgical technique of fascial closure in all median laparotomy cases was a mass closure technique with large bites using 2 looped number 1 PDS sutures (Ethicon, Johnson&Johnson, Norderstedt, Germany).

Furthermore, the radiologic database was sought for preoperative standard contrast enhanced CT scans that were carried out either for staging purposes in malignant diseases or for emergent indications.

The patients were examined in clinical routine using a 128-slice CT scanner (Ingenuity, Philips, Amsterdam, the Netherlands). Intravenous administration of an iodine-based contrast medium (90-mL Imeron 400 MCT, Bracco Imaging Germany GmbH, Konstanz, Germany) was given at a rate of 2–4.0 mL/s via a peripheral venous line. Automatic bolus tracking was performed in the descending aorta with a trigger of 100 Hounsfield units (HU). CT images were acquired in portal venous phase in every case after 70 s delay. Typical imaging parameters were as follows: 100 kVp; 125 mAs; slice thickness, 1 mm; and pitch, 0.9.

The measurements were performed on a clinical PACS workstation (iDS7, Sectra AB, Linköping, Sweden). The 3-dimensional viewer tool was used for the visualization of the linea alba.

**Fig. 1:**
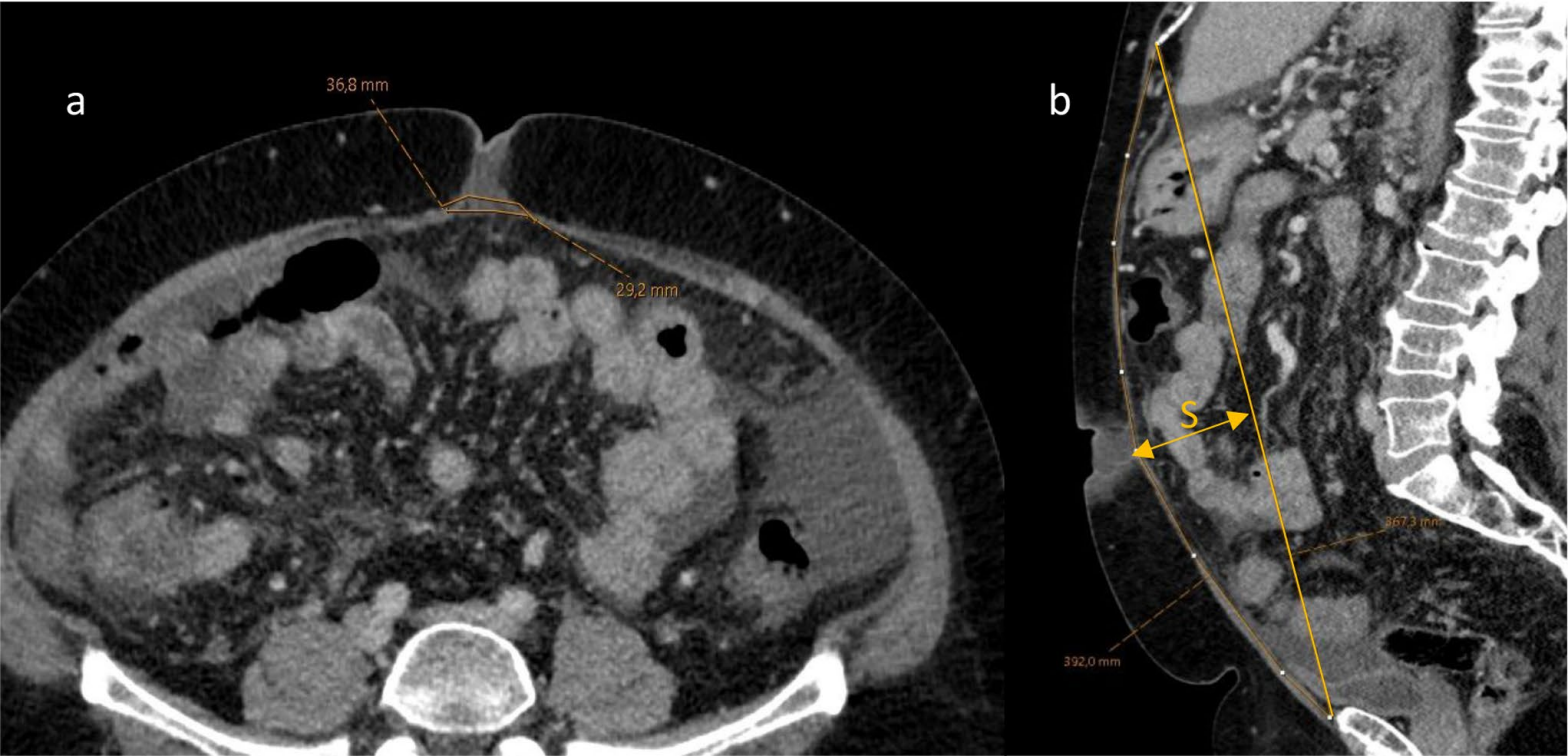
Contrast enhanced CT scan in axial (**a**) and sagittal (**b**) slices with annotations measuring the linea alba width and the length of linea alba. In (**c**) we have highlighted the two lines required for the reconstruction and measurements of the sagitta (S; arrowed line.)

Patients with complete datasets were analyzed using the methods of Geroult et al. [10]. Briefly, the linea alba was visualized in the 3D viewer and distance measurements were performed at predefined locations: total distance of linea alba, sagitta of the linea alba and width measures at six different points along the linea alba as well as the maximal inter rectus distance.

In the original work, patients were compared forming subcohorts according to BMI (three subgroups) and age (four subgroups). As an exhaustive subdivision of the limited number of cases in our cohort would predispose for statistical implausibility, we simplified the groups by subdividing them only by BMI below or over 30 kg/m^2^ and age below and above 60 years.

A control group from the same register was established to compare clinical features of patients with BA to those with a superficial surgical site infection only. The control cohort consisted of patients with a median laparotomy that had been randomly selected from the anonymized data set.

Descriptive statistics were expressed by mean (± standard deviation) for normally distributed continuous variables or as median (interquartile range) in case of no normal distribution.

The univariate analysis included the use of unpaired t-test or the Mann-Whitney-U test for continuous variables and the X^2^-test or the Fisher’s exact test for dichotomous variables.

The Pearson correlation coefficient was used in selected cases of normally distributed correlations.

For the multivariate regression analysis, the binary logistic regression model with backward stepwise inclusion was chosen. The two-sided significance level was set at *p* = 0.05.

The statistical analysis was performed using SPSS 29 (IBM statistics, Ehningen, Germany).

## Results

### Overall cohort

494 patients were included in the register. Of those, 111 had a burst abdomen and 92 had a complete dataset of preoperative CT scans and were further considered for 3D reconstruction analysis.

As fourteen patients did not receive a midline laparotomy, i.e. transverse abdominal incision, they were excluded from the analysis. Furthermore, six patients showed massive abdominal distension or a laparostomy in their CT scan, resulting in implausible measurements or impossibility to properly apply the measurements. Hence, the final subgroup consisted of 72 patients.

The total collective of the burst abdomen group and controls consisted of 72 BA patients and 32 controls, in total 104 patients, being 69 male and 35 female with a mean age of 64.1 (± 14.76) years and a mean BMI of 26.63 (± 5.69) kg/m^2^.

Length of linea alba and sagitta were normally distributed, whereas the rest of the measurements were non-normally distributed. Accordingly, mean and median with the respective t-test or Mann Whitney-test were used. Linea alba had a mean length of 38.26 (± 5.68) cm and a sagitta of 4.35 ± 2.82 cm. The maximum linea alba width showed a median of 5.3 cm (IQR 4.3–7.38). For easier future interpretation of the data, we calculated the correlation between the linea alba and body size (*r* = 0.465 with a *p* < 0.001), linea alba and weight (*r* = 0.66; *p* < 0.001) and BMI (*r* = 0.618; *p* < 0.001).

### Linea alba measurement subgroups

Several subgroup analyses were carried out for age, sex and BMI.

The age-related analysis did not reveal significant differences of the measurements except for the linea alba being shorter above 60 years (40.05 cm (± 5.41 cm) and 37.15 cm (± 5.62 cm); *p* = 0.012).

The sex related subgroup analysis showed significant sex differences with values for male being higher than values for female: lengths of linea alba (*p* < 0.001), sagitta (*p* < 0.001), maximal width of the linea alba (*p* = 0.012), width halfway xyphoid-umbilicus (*p* = 0.024) and width at umbilicus (*p* = 0.012).

Patients with a BMI over 30 kg/m^2^ had significantly higher values in all measurements.

The exact values are depicted in Table 1.

**Table 1:** Shows the values in cm of subgroups according to age, BMI and sex. Length of linea Alba and sagitta are given as mean (± standard deviation), and tested with t-test. The rest of the values are given as median (interquartile range) und tested with Mann-Whitney test

	Overall Cohort	Age < 60y (*n* = 64)	Age > 60y (*n* = 40)	*p*-value	BMI < 30 kg/m^2^ (*n* = 80)	BMI > 30 kg/m^2^ (*n* = 24)	*p*-value	Male (*n* = 69)	Female (*n* = 35)	*p*-value
Length of linea alba	38.26 (± 5.69)	40.05 (± 5.41)	37.15 (± 5.62)	0.012*	36.77 (± 5.33)	43.11 (± 3.85)	< 0.001**	39.48 (± 6.05)	35.71 (± 3.81)	< 0.001**
Sagitta	4.35 (± 2.83)	4.72 (± 2.66)	4.14 (± 2.92)	0.342	3.68 (± 2.53)	6.42 (± 2.72)	< 0.001**	4.94 (± 3.01)	3.1 (± 1.87)	< 0.001**
Width linea alba	5.30 (4.3–7.38)	5.7 (4.5–7.2)	5.2 (4.1–7.7)	0.561	4.8 (4.1–6.3)	8.25 (6.85–11.25)	< 0.001**	6.1 (4.6-8)	4.6 (4.1–6.2)	0.012*
Interrectus distance	4.2 (3.28–5.93)	4.1 (3.2–5.9)	4.3 (3.3–6.3)	0.61	4.1 (3.2–5.03)	6.6 (4.18–8.98)	< 0.001**	4.5 (3.4–6.45)	3.8 (3.1–4.9)	0.085
width half-way xyphoid-umbilicus	3.05 (2-4.63)	2.9 (1.8-4.0)	3.1 (2.1–4.9)	0.284	2.75 (1.8–3.8)	4.6 (3.57–7.48)	< 0.001**	3.3 (2.1–4.9)	2.6 (1.55–3.65)	0.024*
Width 3 cm above umbilicus	3.55 (2.38–5.2)	3.2 (2.1–4.2)	3.7 (2.6–5.3)	0.133	3.15 (2.1–4.45)	5.05 (3.63–7.4)	< 0.001**	3.8 (2.45–5.25)	3.2 (2.25–4.7)	0.35
Width at umbilicus	5.35 (4.3–7.38)	5.7 (4.5–7.2)	5.3 (4.1–7.7)	0.568	4.8 (4.1–6.3)	8.25 (6.85–11.25)	< 0.001**	6.1 (4.6-8)	4.6 (4.1–6.2)	0.012*
Width 2 cm below umbilicus	2.7 (1.8–3.88)	2.8 (1.7–4.1)	2.7 (1.9–3.8)	0.989	2.35 (1.68–3.43)	3.75 (2.85–6.55)	< 0.001**	2.7 (1.85–4.45)	2.4 (1.7–3.25)	0.154
Width half-way umbilicus-pubis	0.8 (0.5–1.4)	0.7 (0.41.5)	0.9 (0.61.4)	0.247	0.8 (0.5–1.4)	0.8 (0.6–1.4)	0.397	0.8 (0.5–1.4)	0.9 (0.6.45)	0.188

### Linea alba measurements in burst abdomen cohort

As main intention of this study, the analysis of the linea alba measurements between the burst abdomen and the controls was carried out in the similar way.

The width of the linea alba was significantly wider in all sites except for halfway umbilicus-pubis in the BA group. The absolute differences were not as pronounced as the differences in the BMI groups but still significant. Additionally, the sagitta was significantly longer in the BA group (4.83 (± 2.93) versus 2.99 (± 1.98); *p* < 0.001), whereas the linea alba total length showed a trend towards longer values in the BA group (38.93 (± 5.77) versus 36.67 (± 5.25); *p* = 0.065). Those measurements are depicted in Table 2.

**Table 2:** Shows the measurements in cm for the burst abdomen and control group. Length of linea Alba and sagitta are given as mean (± standard deviation), and were tested with t-test. The rest of the values are given as median (interquartile range) und tested with Mann-Whitney test

	No BA (*n* = 32)	BA (*n* = 72)	*p*-value
Length of linea alba	36.67 (± 5.25)	38.93 (± 5.77)	0.068
Sagitta	2.99 (± 1.98)	4.83 (± 2.93)	< 0.001**
Width linea alba	4.7 (3.48–6.98)	5.95 (4.6–7.7)	0.014*
interrectus distance	3.75 (2.46-5)	4.45 (3.5–6.45)	0.013*
width half-way xyphoid-umbilicus	2.55 (1.58–3.85)	3.25 (2.15–4.88)	0.022*
Width 3 cm above umbilicus	2.85 (1.98–4.23)	3.75 (2.6–5.3)	0.01*
Width at umbilicus	4.7 (3.48–6.98)	5.95 (4.6–7.7)	0.014*
Width 2 cm below umbilicus	1.95 (1.48–3.13)	2.8 (2.03–4.48)	0.004*
Width half-way umbilicus-pubis	0.7 (0.4–1.33)	0.85 (0.6–1.4)	0.1

### Univariate analysis

The X^2^ test did not show any significant association of age group (*p* = 0.829) or BMI group (*p* = 0.315) with the risk of burst abdomen. Nevertheless, in the BMI > 30 kg/m^2^ 79.2% suffered from BA compared to 66.3% in the BMI < 30 kg/m^2^. Sex was a significant risk factor for BA (*p* = 0.007).

Baseline characteristics of BA group did not differ significantly from the controls with regard to age (63.68 ± 14.04 versus 65.04 ± 16.47 years, *p* = 0.67), BMI (27.16 ± 5.62 versus 25.42 ± 5.73 kg/m^2^, *p* = 0.151), emergency procedures (*p* = 0.089). Male patients suffered significantly more often from burst abdomen (*p* = 0.007). Comorbidities such as cardiovascular diseases, diabetes mellitus, dementia, tumors with or without chemotherapy or chronic inflammatory diseases did not differ significantly.

Of note, differences of comorbidities or clinical conditions in the BA group compared to the control were the following: intestinal resection (*p* = 0.009), liver cirrhosis (*p* = 0.019), intake of immunosuppressants (*p* = 0.02) and revision surgery (*p* = 0.005). If the sagitta was grouped in relation to the overall mean, being higher or lower as the mean, it showed significant differences in BA for the higher sagitta group (*p* = 0.034).

### Multivariate analysis

Finally, the linea alba measurements were entered in the binary logistic regression model together with the statistically apparent parameters mentioned above (intestinal resection, emergency, liver cirrhosis, immunosuppression, need for revision surgery) using the occurrence of BA as dependent variable. Sagitta was the only significant parameter for the prediction of burst abdomen (OR 1.266; 95% CI 1.011–1.585; *p* = 0.04). The factor sex showed a trend with a *p* = 0.074 with an OR 0.392 (95% CI 0.141–1.095), hinting at female sex to be protective against burst abdomen.

**Fig. 2:**
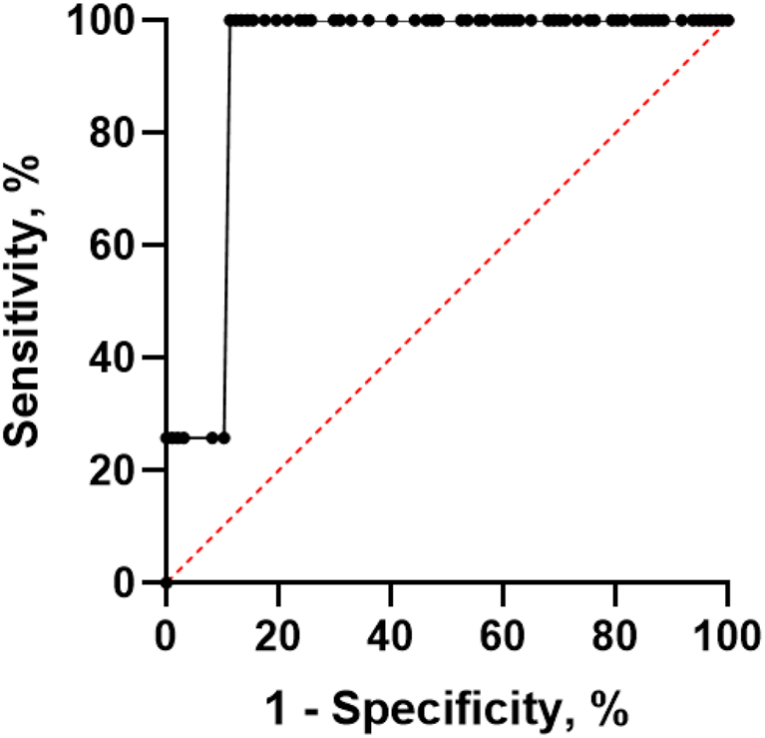
The receiver of operating characteristics curve of the sagittal diameter to predict burst abdomen. The resulting area under the curve is 0.91 (95%CI 0.87;0.96)

The receiver of operating analysis revealed an area under the curve of 0.91 (95%CI 0.87;0.96) for the sagittal length. The threshold of 1.05 cm resulted as the most accurate discriminator in a sensitivity of 100% (95%CI 96.2;100) and a specificity of 88.6% (95%CI 80.8;93.5). The corresponding graph is displayed in Fig. 2.

## Discussion

In the present study, CT- morphometric properties of the linea alba were analyzed and their significance for burst abdomen occurrence were evaluated. Previous studies have shown differences of morphometrics of the abdominal wall among age groups and in relation to BMI [7, 12]. Compared to Gueroult [10], the linea alba in our patients was generally wider and age showed no significant influence. In contrast, we found a gender difference with male patients having a wider linea alba above the umbilicus. Possible structural differences in fiber architecture between men and women have been suggested in the past [13] and may lead to those results. Our data confirmed the influence of the BMI on linea alba measurements stated before [10], as all measurements were significantly wider with patients of BMI over 30 kg/m^2^ compared to those with BMI below 30 kg/m^2^ by the factor of two. However, the sagitta was remarkably longer in obese patients in both study populations (4.8 ± 2 cm [10] and 6.42 ± 2.72 cm in our group), reflecting an increased curvature of the linea alba. As the previous studies were only descriptive morphometric studies evaluating the methodology of CT derived parameters, our data is the first with clinical implication on a surgical outcome, adding clinical importance to these theoretical values.

Miller et al. found out that a higher BMI and longer incision length required more tension to approximate fascial edges of the linea alba during laparotomy. This could reflect the tension on the abdominal wall regarding intraabdominal pressure exerted by intraabdominal obesity [14]. The same group reported, that after component separation for large ventral hernia at the end of the preparation period, the tension needed to approximate the fascial edges was even higher than after primary laparotomy. This additional force would be needed to counteract the retraction of the muscles and fascias during the time of hernia creation [15].

The surgical suture works against the forces of the intraabdominal tissues as well as the tension of the abdominal wall during midline closure after laparotomy. It is known that transverse abdominal incisions are associated with less incisional hernias, even in laparoscopic surgery where typically short incisions are used [16]. Further, Pfannenstiel’s incision is even more favorable than other transverse abdominal incisions [17]. To support those observations, an ex-vivo study reported the best compliance of the linea alba in the longitudinal direction opposed to the compliance in the transverse direction [18]. The compliance is the result of the fibers in the linea alba and their ability to rearrange according to the applied tension. Hence, if all fibers are aligned, the compliance has reached its limit. Additionally, the thickness of the fibers differs along the linea alba [13]. Using an elastic suture material for abdominal wall closure could increase the compliance in the transverse direction, ameliorating postoperative burst abdomen and hernia rate [19].

The law of Laplace reflects the tension of an organ wall, ideally a ball or a cylinder. In a simplified way, the tension of the organ wall (K), in our case the abdominal wall, is dependent on the transmural pressure gradient (P), the radius of the organ (r) and the wall thickness (d).

$$\:K=\frac{P\:\cdot\:r}{d}$$

Even more complex geometrical figures may be used in modelling abdominal wall pressures [20], requiring more complex models. Applying the simplified formula on the abdominal wall, it shows an increase of the abdominal radius leading to a proportional increase of the transmural tension. This condition occurs in cases of increased intraabdominal obesity with a subsequently increased intraabdominal diameter. Hypothetically, an increased tension on the abdominal wall would facilitate burst abdomen, especially in cases of surgical site infection where the fascial tissue is weakened by inflammatory processes. As surrogate, the distance between the vertebral edge and the linea alba on CT scans could be used to predict the risk of burst abdomen [6].

A similar parameter, that evaluates an increased abdominal diameter is the sagitta, suggested in the initial morphometric study [10]. The sagitta displays the curvature of the linea alba in the sagittal plane, forming a rather curved instead of a linear plane. This curvature leads to an increased tension on the linea alba. During regression analysis, the sagitta was the parameter that could be determined as a risk factor for the development of burst abdomen, underlining these theoretical considerations. Our ROC analysis showed a cut-off value of about one centimeter of the sagitta to be already predictive of burst abdomen, which represents only a slight deviation from the linear plane, in the supine position during CT scan. Gueroult et al. stated that some patients had negative sagitta [10] which would reflect the abdomen of a cachectic patient, we have excluded those measurements in a total of five patients, as this influences the calculation of the mean. The sagitta displayed gender and BMI differences, which also could be found in the sex-specific burst abdomen rate. The sagitta was almost twice the length in patients with a BMI higher than 30 kg/m^2^ in our cohort. The sagitta seems to reflect male and female fat tissue distribution types such as intraabdominal rather than subcutaneous obesity, leading to an increased risk of BA in men. Those observations are in line with previous risk scores which have included gender and overweight/BMI as risk factor for BA [2, 4, 5]. In our analysis, BMI did not differ significantly between BA patients and controls and did not pose a risk factor in the multivariate regression. Therefore, we suggest the sagitta instead of BMI as risk factor for BA as the sagitta rather focuses on abdominal obesity.

An increased transmural pressure gradient of the abdominal wall will be present due to an increase in intraabdominal pressure. This usually occurs postoperatively during mobilization, paralytic ileus or coughing. General physiologic intraabdominal pressures have been discussed in a model previously [20]. The values have been deduced by ex-vivo measurements from other studies. Thus, the modelized physiologic pressures reflected in that study may diverge from postoperatively occurring pressures caused by massive bowel distension, i.e. postoperative bowel paralysis, or coughing patterns due to pneumonia or chronic pulmonary disease. Pulmonary conditions could be shown to represent significant risk factors for BA [2–4]. Moreover, in-vivo measurements could demonstrate higher values of intraabdominal pressures by voluntary or reflex cough [21] and even larger increases during daily life and exercising [22], relativizing those theoretical consideration, hinting at even larger pressures which the postoperative abdominal wall has to withstand.

Inversely, the thinner the abdominal wall, the greater the resulting force on the respective abdominal wall segment, requiring more stability by single fibers. The tissue quality and consecutive stability is impaired due to inflammatory processes in surgical site infections (SSI), which also are a risk factor for burst abdomen [23]. Our patient cohort was established from all patients suffering from SSI. Burst abdomen mostly occurs in the cranial part of the laparotomy which may be caused by the transverse tension of the muscles but also thinner architecture of the linea alba [13]. The method of Gueroult did not take into account the thickness of the linea alba. Our data shows an association with rectus diastasis and burst abdomen. Similar results have been published recently in a cohort of emergency laparotomies [24]. While evaluating the images for the possible measurements of the linea alba, several patients had to be excluded, as the abdominal wall was very subtle and thus the measurements of the linea alba were largely implausible. Hypothetically, those patients were at an even increased risk of burst abdomen due to the very thin abdominal wall and consequently would need preemptive tissue reinforcement during abdominal wall closure [25], which has been suggested recently to improve hernia rates.

### Limitations

Our study has some limitations that have to be discussed. First, the study population consisted of a retrospectively assembled and preselected cohort of patients suffering from postoperative wound infections. The measurements of the linea alba in our cohort were mostly not normally distributed, as has been reported by Gueroult [10], representing a large variance. Therefore, non-parametric tests were used. Thus, a much larger cohort would yield normally distributed values, but in recent years, no comparable larger cohorts of burst abdomen patients have been published. Further, measuring the linea alba width in patients with very thin abdominal wall may comprise larger variance as the solution of the images could limit proper delineation of the linea alba. Moreover, there might be some interreader variability in the investigated measurements. There is definite need for further validation of the investigated measurements in regard of stability between different CT scanners, readers, 3D viewers and if the measurements could be transferred to magnetic-resonance imaging examinations.

## Conclusion

The results from this study suggest the linea alba curvature reflected by the sagitta in CT scans as a CT-morphometric risk factor for postoperative burst abdomen. Furthermore, we could confirm the findings previously presented for CT derived 3D morphometric data on the linea alba. Our data adds a clinical implication on 3D morphometrics and another dimension for preoperative risk assessment with regard to burst abdomen.
